# Relationship between obesity indices and cognitive function in Japanese men: A cross-sectional study

**DOI:** 10.1371/journal.pone.0332595

**Published:** 2025-10-23

**Authors:** Satoshi Matsuno, Yuji Ozeki, Sayaka Kadowaki, Sayuki Torii, Keiko Kondo, Naoko Miyagawa, Azusa Shima, Mizuki Ohashi, Itsuko Miyazawa, Hiroyoshi Segawa, Takashi Hisamatsu, Aya Kadota, Katsuyuki Miura

**Affiliations:** 1 Department of Psychiatry, Shiga University of Medical Science, Shiga, Japan; 2 Department of Physical Therapy, Osaka Yukioka College of Health Science, Osaka, Japan; 3 NCD Epidemiology Research Center, Shiga University of Medical Science, Shiga, Japan; 4 Department of Public Health, Shiga University of Medical Science, Shiga, Japan; 5 Department of Preventive Medicine and Public Health, Keio University School of Medicine, Tokyo, Japan; 6 Department of Clinical Nursing, Shiga University of Medical Science, Shiga, Japan; 7 Occupational Health Care Office, Heiwado Co., Shiga, Japan; 8 Department of Medicine, Shiga University of Medical Science, Shiga, Japan; 9 Hirosemirai Clinic, Miyazaki, Japan; 10 Department of Public Health, Okayama University Graduate School of Medicine, Dentistry, and Pharmaceutical Sciences, Okayama, Japan; Università degli Studi di Milano, ITALY

## Abstract

We aimed to investigate the associations among various obesity indices, including visceral (VAT) and subcutaneous adipose tissue (SAT), and cognitive function in community-dwelling Japanese men. This population-based cross-sectional study used data of 853 men who participated in the follow-up examinations of the Shiga Epidemiological Study of Subclinical Atherosclerosis. Among them, we analyzed data of 776 men who completed the Cognitive Abilities Screening Instrument (CASI) and had abdominal VAT and SAT areas measured using computed tomography. The VAT-to-SAT ratio (VSR) was calculated; participants were categorized into VSR quartiles. Using analysis of covariance, we computed crude and adjusted means of the CASI total and domain scores across VSR quartiles, adjusting for potential confounders. No significant differences were observed in total CASI scores among body mass index, VAT, or SAT quartiles. However, in the multivariable-adjusted model, participants in the lowest VSR quartile (Q1) had significantly lower CASI total scores than those in the third quartile (Q3) (Q1: 89.5, Q3: 90.9). Low VSR was independently associated with lower cognitive function in a community-based sample of middle-aged and older Japanese men. In summary, VSR may be associated with cognitive function in Japanese men, highlighting the importance of fat distribution in cognitive health and highlighting VSR as a useful indicator.

## Introduction

Previous studies investigating the relationship between obesity indicators and cognitive function have reported that a higher body mass index (BMI) is associated with lower cognitive function and an increased risk of dementia [1–5] and that greater area of visceral adipose tissue (VAT) is linked to lower cognitive function and an increased risk of dementia [6–10]. Visceral fat accumulation affects the brain structure through insulin resistance, inflammation, and vascular injury, which may be related to cognitive decline [11,12].

Conversely, some studies have not found a relationship between VAT and cognitive function [13–15], whereas others have shown that increased subcutaneous adipose tissue (SAT) is associated with decreased cognitive function [13,15,16] and that low BMI increases the risk of dementia [17]. Therefore, no consensus exists on the relationship between obesity indicators and cognitive function.

In recent years, studies have reported that VAT and the VAT-to-SAT ratio (VSR) are associated with amyloid pathology [18], drawing attention to the balance between VAT and SAT. However, the association between VSR and cognitive function in Asian populations has not been thoroughly investigated. Therefore, in this study we investigated the association between various obesity indices (including VAT and SAT, evaluated using computed tomography [CT]) and cognitive function in a community-based sample of Japanese men.

## Methods

### Study design and participants

This population-based cross-sectional study used data from the Shiga Epidemiological Study of Subclinical Atherosclerosis (SESSA), The SESSA is an observational cohort study with follow-up from 2009 to 2014. We conducted the SESSA to investigate subclinical atherosclerosis and its determinants in a sample of Japanese residents, and recorded the participants’ Cognitive Abilities Screening Instrument (CASI) scores. Enrollment methods for the SESSA have been reported previously [19–21]. In summary, from 2006 to 2008 (baseline), we randomly selected and invited 2,379 Japanese men (aged 40–79 years, from Kusatsu City, Shiga) to participate, using the Basic Residents’ Register. In total, 1,094 men agreed to participate in the baseline examination and 853 men participated in the follow-up study, with participation rates of 46% and 78%, respectively. For our current study, we excluded participants of the SESSA who did not complete the CASI at the follow-up examination (n = 39) and those with missing CT data on areas of SAT and VAT (n = 38), leaving 776 men for the final analyses.

### Ethics declaration

This study was approved by the Institutional Review Board of the Shiga University of Medical Science (approval number G2008-061). Written informed consent was obtained from all participants. This study does did not required trial registration owing to its observational design.

### Measurements

Data on sociodemographic and lifestyle factors, including history of alcohol intake (never, past, and current), smoking (never, past, and current), and physical exercise (number of days per week of leisure-time physical activity), were collected using a self-administered questionnaire. Medical histories of hypertension, diabetes, and dyslipidemia were recorded along with corresponding medication use.

Blood pressure was also measured using an automated sphygmomanometer (BP-8800; Omron HealthCare Co., Ltd., Tokyo, Japan). The mean of two consecutive measurements taken on the right arm, with participants seated after a 5-min rest, was used as the blood pressure value. We defined hypertension as systolic blood pressure ≥140 mmHg, diastolic blood pressure ≥90 mmHg, and use of antihypertensive medication.

Blood samples were collected to determine fasting blood sugar, hemoglobin A1c, and serum lipid levels. Diabetes mellitus was defined as hemoglobin A1c level ≥6.5% based on the National Glycohemoglobin Standardization Program [22], fasting blood glucose level ≥126 mg/dL, or use of antidiabetic medications. Total cholesterol and triglyceride levels were measured using enzymatic assays, and high-density lipoprotein cholesterol levels were assessed using a direct method. Lipid measurements were standardized according to the protocol of the United States Centers for Disease Control and Prevention/Cholesterol Reference Method Laboratory Network. Low-density lipoprotein cholesterol level was estimated using the Friedewald formula and was treated as missing if the triglyceride level was > 400 mg/dL [23]. Dyslipidemia was defined as low-density lipoprotein cholesterol level ≥3.6 mmol/L (140 mg/dL), high-density lipoprotein cholesterol level <1.0 mmol/L (40 mg/dL), triglyceride level ≥150 mg/dL, or use of lipid-lowering medication.

### Cognitive function

Cognitive function was evaluated based on the participants’ performance on the CASI (version J-1.0). The CASI is a comprehensive measure of intellectual function comprising 25 questions [24]. The CASI scores range from 0 to 100, with higher scores indicating better cognitive function. A CASI score of 82–100 falls in the good range, 74–81 within the intermediate range, and below 74 within the poor range [25]. In this study, we analyzed the overall cognitive function score and each domain score of the CASI. The CASI domains include attention (score range: 0–8), concentration (score range: 0–10), orientation (score range: 0–18), short-term memory (score range: 0–12), long-term memory (score range: 0–10), language ability (score range: 0–10), visuoconstruction (score range: 0–10), list generation fluency (score range: 0–10), and abstraction and judgment (score range: 0–12) [24].

### Obesity indexes

Body weight, height, and body fat percentage were measured with the participants wearing light clothing without shoes. BMI was calculated as weight (kg) divided by height squared (m^2^). Waist circumference was measured twice at the level of the umbilicus, and the average value was calculated. Hip circumference was measured twice at the level of maximal hip protrusion, and the average value was calculated. The waist-to-hip ratio was calculated as waist circumference (cm) divided by hip circumference (cm). The waist-to-height ratio was calculated as waist circumference (cm) divided by height (cm).

The areas of VAT and SAT were assessed using CT (MDCT, Aquilion-16; Toshiba Medical Systems, Tochigi, Japan; slice thickness, 7 mm). Abdominal VAT was defined as the fat enclosed by the inner aspect of the abdominal wall. Abdominal SAT was defined as fat outside the outer aspects of the abdominal wall, excluding that within the muscular fascia. While the participants were placed in the supine position, serial CT images were obtained using a protocol similar to that described previously [26]. A single CT image of the L4–L5 vertebral space was selected to estimate the areas of the VAT and SAT. Adipose tissue was identified as having attenuation between −190 and −30 Hounsfield units, combined with anatomical interpretation by the reader. The inner and outer aspects of the abdominal walls were manually tracked, and the respective areas were calculated using image analysis software (Slice Omatic; Tomovision, Montreal, Canada). All CT images were analyzed at the Shiga University of Medical Science by experienced physician-researchers who were blinded to the characteristics of the participants in the current study.

The area of total adipose tissue was calculated as the sum of the areas of VAT and SAT. The VSR was calculated by dividing the area of VAT by the area of SAT. CT images were analyzed at the Shiga University of Medical Science by a trained physician who was blinded to the participants’ characteristics.

### Statistical analysis

Participant characteristics were presented using descriptive statistics. Age-adjusted Pearson’s correlation coefficients were calculated. Analysis of covariance was used to compare mean CASI scores for each obesity index quartile category. Crude and four multivariable-adjusted models were used. In Model 1, we adjusted for age and years of education. In Model 2, Model 1 was further adjusted for BMI. In Model 3, Model 2 was further adjusted for history of smoking, alcohol intake, and physical exercise. In Model 4, Model 3 was adjusted for the prevalence of hypertension, diabetes, and dyslipidemia. The same analysis was performed for the domain-specific cognitive functions. Bonferroni tests were used for post-hoc comparisons between the quartiles of each obesity index.

Statistical analyses were conducted using the SPSS Statistics (version 28.0) software (IBM Corp., Tokyo, Japan). Statistical significance was set at P values <0.05.

## Results

The demographic and health characteristics of the study participants are shown in Table 1. Among the 776 men included in the study, the mean age was 68.4 years, and the mean years of education was 12.7 years. In 92.8% of the study participants (720/776), the CASI score was within the acceptable range (CASI score: 82–100). In 5.7% of the study participants (44/776), the CASI score was in the intermediate range (74–81). In 1.5% of the study participants (12/776), the CASI score was in the poor range (<74). The mean area of VAT, area of SAT, and VSR for participants in this study were 118.7 cm^2^, 116.3 cm^2^, and 1.06, respectively.

**Table 1:** Demographics of participants (776 men, 2009–2014, Shiga, Japan).

Variables	Mean ± SD or %
Age, years	68.4 ± 8.0
Education, years	12.7 ± 2.4
Smoking, %	
Current	21.5
Past	59.4
Never	19.1
Drinking, %	
Current	79.3
Past	5.0
Never	15.7
Hypertension, %	58.9
Diabetes, % n = 773	23.7
Dyslipidemia, % n = 773	56.4
Total CASI score	90.7 ± 5.8
Height, cm	165.8 ± 5.9
Weight, kg	64.3 ± 9.7
Body mass index, kg/m^2^	23.4 ± 3.0
Waist circumference, cm	85.3 ± 8.2
Hip circumference, cm	92.0 ± 5.3
Waist-to-hip ratio	0.93 ± 0.06
Waist-to-height ratio	0.52 ± 0.05
Body fat percentage, %	20.6 ± 6.9
VAT, cm^2^	118.7 ± 55.6
SAT, cm^2^	116.3 ± 51.1
TAT, cm^2^	234.9 ± 96.9
VAT-SAT ratio	1.06 ± 0.41

CASI, Cognitive Abilities Screening Instrument; SD, standard deviation; VAT, area of abdominal visceral adipose tissue; SAT, area of abdominal subcutaneous tissue; TAT, total abdominal visceral adipose tissue.

Age-adjusted Pearson’s correlation coefficients among the obesity indices are shown in Table 2. VSR showed a moderate positive correlation with VAT (r = 0.506) and an inverse correlation with SAT (r = −0.228). No significant correlation was observed between VSR and BMI (r = −0.014).

**Table 2:** Age-adjusted Pearson’s correlation coefficients among obesity indices (776 men, 2009–2014, Shiga, Japan).

	BMI	WC	HC	WHR	WtHR	BFP	VAT	SAT	TAT
WC	0.884								
HC	0.818	0.822							
WHR	0.631	0.819	0.351						
WtHR	0.899	0.945	0.725	0.823					
BFP	0.591	0.613	0.504	0.498	0.613				
VAT	0.721	0.799	0.630	0.678	0.768	0.533			
SAT	0.846	0.861	0.771	0.637	0.843	0.575	0.650		
TAT	0.860	0.912	0.767	0.725	0.885	0.609	0.917	0.900	
VSR	−0.014^a^	0.068^b^	−0.051^c^	0.164	0.058^d^	0.040^e^	0.506	−0.228	0.171

BMI, body mass index; WC, waist circumference; HC, hip circumference; WHR, waist-to-hip ratio; WtHR, waist-to-height ratio; BFP, body fat percentage; VAT, area of abdominal visceral adipose tissue; SAT, area of abdominal subcutaneous tissue; TAT, total abdominal visceral adipose tissue; VSR, VAT-to-SAT ratio.

Unless otherwise specified, all P < 0.001.

^a^P = 0.702, ^b^P = 0.058, ^c^P = 0.159, ^d^P = 0.104, ^e^P = 0.266.

The crude and adjusted means of the total CASI scores according to VAT quartile categories are shown in Table 3. No significant differences were observed in total CASI scores among the VAT quartiles in any of the models. The crude and adjusted means of the total CASI scores according to the VSR quartile categories are shown in Table 4. The CASI scores were significantly lower in Q1 than in Q3.

**Table 3:** Crude and adjusted means of CASI total scores according to VAT quartiles.

	VAT							
	Q1 (n = 195)		Q2 (n = 190)		Q3 (n = 198)		Q4 (n = 193)	
	(0.8–80.1)		(80.5–116.1)		(116.7–151.0)		(151.1–311.2)	
Models	Mean	95% CI	Mean	95% CI	Mean	95% CI	Mean	95% CI
Crude	89.9	89.1–90.7	91.3	90.5–92.2	90.8	90.0–91.6	90.7	89.9–91.5
Model 1	90.5	89.7–91.2	91.0	90.2–91.7	90.6	89.9–91.3	90.8	90.1–91.5
Model 2	90.3	89.5–91.1	90.9	90.2–91.6	90.6	89.9–91.3	91.0	90.1–91.8
Model 3	89.8	88.9–90.8	90.4	89.5–91.3	90.1	89.1–91.0	90.5	89.4–91.5
Model 4	89.7	88.8–90.7	90.3	89.4–91.2	90.0	89.0–91.0	90.4	89.3–91.5

Data are presented for n = 776 men, 2009–2014, Shiga, Japan.

CASI, Cognitive Abilities Screening Instrument; VAT, area of abdominal visceral adipose tissue; CI, confidence interval.

Model 1 was adjusted for age and years of education.

Model 2 was adjusted for variables in Model 1 plus body mass index.

Model 3 was adjusted for the variables in Model 2 plus smoking (never, past, current), drinking (never, past, current), and exercise (number of days per week of leisure-time physical activity).

Model 4 was adjusted for the variables in Model 3 plus hypertension (yes or no), diabetes (yes or no), and dyslipidemia (yes or no).

No significant differences were observed among VAT quartiles.

**Table 4:** Crude and adjusted means of total CASI scores according to the VSR quartiles.

	VSR							
	Q1 (n = 194)		Q2 (n = 194)		Q3 (n = 194)		Q4 (n = 194)	
	(0.14–0.75)		(0.75–1.00)		(1.00–1.30)		(1.31–2.91)	
Models	Mean	95% CI	Mean	95% CI	Mean	95% CI	Mean	95% CI
Crude	90.2	89.4–91.0	90.7	89.9–91.5	91.7	90.9–92.5	90.2	89.4–91.0
Model 1	90.1*	89.4–90.8	90.7	90.0–91.4	91.5	90.8–92.2	90.6	89.9–91.3
Model 2	90.1*	89.4–90.8	90.7	90.0–91.4	91.5	90.8–92.2	90.6	89.9–91.3
Model 3	89.6*	88.7–90.4	90.2	89.3–91.1	91.0	90.1–91.9	90.0	89.1–91.0
Model 4	89.5*	88.6–90.4	90.1	89.1–91.0	90.9	90.0–91.9	90.0	89.0–90.9

Data are presented for n = 776 men, 2009–2014, Shiga, Japan.

CASI, Cognitive abilities screening instrument; VSR, area of abdominal visceral adipose tissue-to-area of abdominal subcutaneous tissue ratio; CI, confidence interval.

Model 1 was adjusted for age and years of education.

Model 2 was adjusted for variables in Model 1 plus body mass index.

Model 3 was adjusted for the variables in Model 2 plus smoking (never, past, current), drinking (never, past, current), and exercise (number of days per week of leisure-time physical activity).

Model 4 was adjusted for the variables in Model 3 plus hypertension (yes or no), diabetes (yes or no), and dyslipidemia (yes or no).

*Significantly lower than Q3 according to the Bonferroni tests (P < 0.05).

No significant differences were observed in the total CASI scores among the quartile categories of the other obesity indicators (S1–S8 Tables). Moreover, no significant differences were observed in CASI domain scores among the VSR quartiles in any model (S9 Table).

## Discussion

This study investigated the association between various obesity indices, including VAT, SAT, and VSR, and cognitive function in a community-based sample of Japanese men.

No significant differences were observed in the total CASI scores across the VAT and SAT quartiles. However, in the VSR quartiles, the adjusted mean total CASI scores were significantly lower in the lowest quartile (Q1) than in the third quartile (Q3). Notably, this result was observed after adjusting for confounding factors such as educational history, BMI, and other cardiovascular risk factors. Our findings indicate the importance of focusing on the VSR in Japanese men with relatively lower obesity.

Previous studies have reported that people with increased VAT area have a higher risk of developing dementia and that VAT is associated with a decline in cognitive function [6–10]. However, other studies have reported no relationship between VAT and cognitive function [13–15]. Our results in community-dwelling Japanese men showed no significant differences in cognitive function between the VAT quartiles, supporting the results of previous studies [13–15]. It remains unclear why greater area of VAT was associated with an increased risk of dementia and cognitive decline in some studies, whereas other studies showed no association between VAT and dementia risk or cognitive decline. Possible reasons for these inconsistent findings include the use of different methods to evaluate cognitive function, differences in participants’ obesity levels between the studies, and that previous studies on Asian individuals reported a lower participants’ average BMI [9,10] compared with that of previous studies on Western individuals [6,14].

A previous study [18] that investigated the relationship of VSR with positron emission tomography-determined amyloid and tau accumulation in default mode network areas and magnetic resonance imaging (MRI)-determined brain volume and cortical thickness in Alzheimer’s disease-signature areas reported that individuals with high VSR had severe amyloid pathology in the right precuneus cortex, and that visceral obesity, insulin resistance, and reduced cortical thickness in the Alzheimer’s disease-signature areas were associated with high amyloid pathology. These results were in contrast to those of the present study. This difference in results may be attributed to the fact that the mean BMI of the subjects in the previous study [18] was 32.3 kg/m^2^, higher than that of the Japanese men in the present study, where the obesity rate was low. In addition, the volume of adipose tissue was calculated using MRI in the previous study [18], whereas the area was calculated using CT in the present study; this may have affected the results.

One potential mechanism for the association between a lower VSR and cognitive decline may be the adverse effects on nerve function due to the excessive accumulation of SAT relative to VAT.

A previous study investigating the relationship between abdominal fat and cognitive function using CT reported no significant association between VAT and cognitive function [13]. However, the highest tertile of SAT was associated with significantly lower cognitive function compared with the lowest tertile in older adult men [13]. Previous studies on excess SAT accumulation have reported that individuals with high area of SAT have lower axon density in white matter than individuals with low area of SAT [27]. Individuals with high area of SAT are reported to have significantly higher levels of amyloid burden than individuals with low area of SAT, as measured using amyloid positron emission tomography [27]. Considering these findings, subcutaneous fat obesity caused by excessive accumulation of SAT relative to VAT may lead to a decline in cognitive function through reduced axon density and increased amyloid accumulation.

Other potential reasons for the association between decreased VSR and cognitive decline are the lower capacity of Asians to store energy in subcutaneous fat [28], and the possibility that reduced VSR decreases adiponectin secretion from adipocytes. The imbalance between VAT and SAT reduces adiponectin secretion, which may adversely affect cognitive function.

The results of this study showed that there was a significant difference in the total CASI score between VSR quartiles Q1 and Q3, but not between VSR quartiles Q1 and Q4. One reason for this finding is that, although no statistically significant difference was observed, VSR quartile Q4 tended to have a lower CASI total score. This suggests that in Japanese men, who have a relatively low prevalence of obesity, both low and high VSR can lead to a decline in cognitive function. Thus, maintenance of an appropriate VSR may play an important role in the maintenance of cognitive function.

Although there was a significant difference in the CASI total score between VSR quartiles in the present study, there was no significant difference in each CASI domain score between VSR quartiles, probably because the CASI total score ranges from 0 to 100 points, while the CASI domain scores range from 0 to 8 points or 10, 12, and 18 points. This narrows the range of domain scores and the difference in average values relative to the total score, making it difficult to observe statistically significant differences in multiple comparisons.

However, to the best of our knowledge, no study has assessed VSR using CT and investigated its association with cognitive function; therefore, this is the first study to investigate the relationship between cognitive function and detailed obesity indices, including not only BMI and waist circumference but also VSR assessed using CT. Therefore, our findings add to the literature by confirming the association between the VSR quartiles and cognitive function in Asian populations with lower obesity rates.

This study had some limitations. First, because this was a cross-sectional study, a causal relationship between VSR and cognitive function could not be demonstrated. Second, this study only included men; therefore, the results cannot be extrapolated to women. Third, because few participants had cognitive impairment, the applicability of the results may be limited to mild cognitive decline. Finally, the results of this study were obtained from a population with a relatively low prevalence of obesity; thus, the results may not apply to populations with a high prevalence of obesity.

## Conclusion

This study demonstrated that a lower VSR was independently associated with lower cognitive function in a community-based sample of relatively lean Japanese men. These results suggest that in Japanese men, attention should be paid to VSR rather than VAT and SAT separately. Therefore, further research is required to determine the relationship between obesity indices and cognitive function.

### Data reporting

The datasets generated and/or analyzed during the current study are not publicly available due to privacy or ethical reasons.

## Supporting information

S1 Table Crude and adjusted means of the total CASI scores according to the body mass index quartiles (776 men, 2009–2014, Shiga, Japan). (DOCX)

S2 Table Crude and adjusted means of the total CASI scores according to the waist circumference quartiles (776 men, 2009–2014, Shiga, Japan). (DOCX)

S3 Table Crude and adjusted means of the total CASI scores according to hip circumference quartiles (776 men, 2009–2014, Shiga, Japan). (DOCX)

S4 Table Crude and adjusted means of the total CASI scores according to waist-to-hip ratio quartiles (776 men, 2009–2014, Shiga, Japan). (DOCX)

S5 Table Crude and adjusted means of the total CASI scores according to waist-to-height ratio quartiles (776 men, 2009–2014, Shiga, Japan). (DOCX)

S6 Table Crude and adjusted means of the total CASI scores according to body fat percentage quartiles (776 men, 2009–2014, Shiga, Japan). (DOCX)

S7 Table Crude and adjusted means of the total CASI scores according to SAT quartiles (776 men, 2009–2014, Shiga, Japan). (DOCX)

S8 Table Crude and adjusted means of the total CASI scores according to TAT quartiles (776 men, 2009–2014, Shiga, Japan). (DOCX)

S9 Table Adjusted means of CASI domain scores according to VSR quartiles (n = 776, 2009–2014, Shiga, Japan). (DOCX)
