# Supplementary material for: Relationship between obesity indices and cognitive function in Japanese men: A cross-sectional study
Source: PLoS One. 2025 Oct 23;20(10):e0332595. doi: 10.1371/journal.pone.0332595 (PMC12548842; doi:10.1371/journal.pone.0332595)
Supplement: S1 Table — (DOCX) [file pone.0332595.s001.docx]

**Supporting Information**

S1 Table. Crude and adjusted means of the total CASI scores according to the body mass index quartiles (776 men, 2009–2014, Shiga, Japan)

|  | Body mass index | | | | | | | |
| --- | --- | --- | --- | --- | --- | --- | --- | --- |
|  | Q1 (n = 196) | | Q2 (n = 194) | | Q3 (n = 194) | | Q4 (n = 192) | |
|  | (14.8–21.5) | | (21.5–23.3) | | (23.3–25.1) | | (25.1–37.2) | |
| Models | Mean | 95% CI | Mean | 95% CI | Mean | 95% CI | Mean | 95% CI |
| Crude | 90.4 | 89.6–91.2 | 90.2 | 89.4–91.0 | 91.5 | 90.7–92.3 | 90.6 | 89.8–91.5 |
| Model 1 | 90.7 | 90.0–91.4 | 90.4 | 89.6–91.1 | 91.5 | 90.8–92.2 | 90.2 | 89.5–90.9 |
| Model 2 | 90.2 | 89.4–91.1 | 89.8 | 89.0–90.7 | 90.9 | 90.0–91.8 | 89.7 | 88.8–90.6 |
| Model 3 | 90.1 | 89.2–91.1 | 89.7 | 88.8–90.7 | 90.9 | 89.9–91.8 | 89.7 | 88.8–90.6 |

CASI, Cognitive Abilities Screening Instrument; CI, confidence interval.

Model 1 was adjusted for age and years of education.

Model 2 was adjusted for the variables in Model 1 plus smoking (never, past, or current), drinking (never, past, or current), and exercise (number of days per week of leisure-time physical activity).

Model 3 was adjusted for the variables in Model 2 plus hypertension (yes or no), diabetes (yes or no), and dyslipidemia (yes or no).

No significant differences were observed among body mass index quartiles.
