# Supplementary material for: Relationship between obesity indices and cognitive function in Japanese men: A cross-sectional study
Source: PLoS One. 2025 Oct 23;20(10):e0332595. doi: 10.1371/journal.pone.0332595 (PMC12548842; doi:10.1371/journal.pone.0332595)
Supplement: S2 Table — (DOCX) [file pone.0332595.s002.docx]

**Supporting Information**

S2 Table. Crude and adjusted means of the total CASI scores according to the waist circumference quartiles (776 men, 2009–2014, Shiga, Japan)

|  | Waist circumference | | | | | | | |
| --- | --- | --- | --- | --- | --- | --- | --- | --- |
|  | Q1 (n = 156) | | Q2 (n = 158) | | Q3 (n = 153) | | Q4 (n = 156) | |
|  | (58.2–80.1) | | (80.2–85.2) | | (85.3–90.7) | | (90.7–115.0) | |
| Models | Mean | 95% CI | Mean | 95% CI | Mean | 95% CI | Mean | 95% CI |
| Crude | 90.7 | 89.9–91.5 | 90.7 | 89.9–91.5 | 90.6 | 89.7–91.4 | 90.8 | 90.0–91.6 |
| Model 1 | 90.8 | 90.0–91.5 | 90.9 | 90.2–91.6 | 90.6 | 89.9–91.3 | 90.6 | 89.9–91.3 |
| Model 2 | 90.2 | 89.3–91.1 | 90.3 | 89.4–91.2 | 90.0 | 89.1–90.9 | 90.1 | 89.2–91.0 |
| Model 3 | 90.1 | 89.2–91.0 | 90.2 | 89.3–91.1 | 89.9 | 88.9–90.9 | 90.1 | 89.1–91.0 |

CASI, Cognitive Abilities Screening Instrument; CI, confidence interval.

Model 1 was adjusted for age and years of education.

Model 2 was adjusted for the variables in Model 1 plus smoking (never, past, or current), drinking (never, past, or current), and exercise (number of days per week of leisure-time physical activity).

Model 3 was adjusted for the variables in Model 2 plus hypertension (yes or no), diabetes (yes or no), and dyslipidemia (yes or no).

No significant differences were observed among waist circumference quartiles.
