# Supplementary material for: Relationship between obesity indices and cognitive function in Japanese men: A cross-sectional study
Source: PLoS One. 2025 Oct 23;20(10):e0332595. doi: 10.1371/journal.pone.0332595 (PMC12548842; doi:10.1371/journal.pone.0332595)
Supplement: S8 Table — (DOCX) [file pone.0332595.s008.docx]

**Supporting Information**

S8 Table. Crude and adjusted means of the total CASI scores according to TAT quartiles (776 men, 2009–2014, Shiga, Japan)

|  | TAT | | | | | | | |
| --- | --- | --- | --- | --- | --- | --- | --- | --- |
|  | Q1 (n = 194) | | Q2 (n = 194) | | Q3 (n = 194) | | Q4 (n = 194) | |
|  | (4.5–170.0) | | (170.3–232.1) | | (232.5–295.4) | | (296.2–571.5) | |
| Models | Mean | 95% CI | Mean | 95% CI | Mean | 95% CI | Mean | 95% CI |
| Crude | 90.0 | 89.2–90.8 | 90.8 | 90.0–91.6 | 91.1 | 90.3–91.9 | 90.9 | 90.1–91.7 |
| Model 1 | 90.4 | 89.7–91.2 | 90.9 | 90.2–91.6 | 90.8 | 90.1–91.5 | 90.7 | 90.0–91.4 |
| Model 2 | 90.2 | 89.3–91.1 | 90.8 | 90.1–91.5 | 90.8 | 90.1–91.5 | 91.0 | 90.0–91.9 |
| Model 3 | 89.8 | 88.7–90.8 | 90.3 | 89.4–91.2 | 90.3 | 89.4–91.2 | 90.5 | 89.3–91.6 |
| Model 4 | 89.7 | 88.6–90.7 | 90.2 | 89.3–91.1 | 90.2 | 89.2–91.2 | 90.4 | 89.3–91.6 |

CASI, Cognitive Abilities Screening Instrument; TAT, total abdominal adipose tissue area; CI, confidence interval.

Model 1 was adjusted for age and years of education.

Model 2 was adjusted for variables in Model 1 plus body mass index.

Model 3 was adjusted for the variables in Model 2 plus smoking (never, past, current), drinking (never, past, current), and exercise (number of days per week of leisure-time physical activity).

Model 4 was adjusted for the variables in Model 3 plus hypertension (yes or no), diabetes (yes or no), and dyslipidemia (yes or no).

No significant differences were observed among TAT quartiles.
