# Supplementary material for: Relationship between obesity indices and cognitive function in Japanese men: A cross-sectional study
Source: PLoS One. 2025 Oct 23;20(10):e0332595. doi: 10.1371/journal.pone.0332595 (PMC12548842; doi:10.1371/journal.pone.0332595)
Supplement: S9 Table — (DOCX) [file pone.0332595.s009.docx]

**Supporting Information**

S9 Table. Adjusted means of CASI domain scores according to VSR quartiles (n = 776, 2009–2014, Shiga, Japan)

|  |  | VSR | | | | | | | |
| --- | --- | --- | --- | --- | --- | --- | --- | --- | --- |
|  |  | Q1 (n = 194) | | Q2 (n = 194) | | Q3 (n = 194) | | Q4 (n = 194) | |
|  | Models | Mean | 95% CI | Mean | 95% CI | Mean | 95% CI | Mean | 95% CI |
| Attention |  |  |  |  |  |  |  |  |  |
|  | Model 1 | 6.88 | 6.75–7.02 | 6.95 | 6.81–7.09 | 7.00 | 6.86–7.14 | 6.90 | 6.76–7.03 |
|  | Model 4 | 6.82 | 6.64–6.99 | 6.86 | 6.68–7.04 | 6.94 | 6.76–7.12 | 6.83 | 6.65–7.02 |
| Concentration |  |  |  |  |  |  |  |  |  |
|  | Model 1 | 8.78 | 8.58–8.97 | 8.97 | 8.77–9.16 | 8.86 | 8.66–9.05 | 9.12 | 8.93–9.32 |
|  | Model 4 | 8.72 | 8.47–8.97 | 8.89 | 8.63–9.15 | 8.82 | 8.56–9.07 | 9.08 | 8.82–9.34 |
| Orientation |  |  |  |  |  |  |  |  |  |
|  | Model 1 | 17.53 | 17.40–17.67 | 17.76 | 17.63–17.90 | 17.75 | 17.61–17.88 | 17.66 | 17.53–17.80 |
|  | Model 4 | 17.49 | 17.32–17.66 | 17.70 | 17.52–17.88 | 17.69 | 17.51–17.87 | 17.59 | 17.41–17.78 |
| Long-term memory |  |  |  |  |  |  |  |  |  |
|  | Model 1 | 9.95 | 9.90–9.99 | 9.95 | 9.91–9.99 | 9.94 | 9.90–9.99 | 9.93 | 9.88–9.97 |
|  | Model 4 | 9.95 | 9.89–10.01 | 9.96 | 9.90–10.02 | 9.95 | 9.89–10.01 | 9.94 | 9.88–10.00 |
| Short-term memory |  |  |  |  |  |  |  |  |  |
|  | Model 1 | 9.18 | 8.91–9.45 | 9.37 | 9.10–9.64 | 9.52 | 9.25–9.79 | 9.30 | 9.03–9.57 |
|  | Model 4 | 8.97 | 8.62–9.31 | 9.13 | 8.77–9.49 | 9.31 | 8.96–9.67 | 9.05 | 8.68–9.41 |
| Language |  |  |  |  |  |  |  |  |  |
|  | Model 1 | 9.83 | 9.75–9.91 | 9.73 | 9.65–9.81 | 9.86 | 9.78–9.94 | 9.83 | 9.75–9.91 |
|  | Model 4 | 9.81 | 9.71–9.91 | 9.71 | 9.60–9.81 | 9.84 | 9.74–9.94 | 9.81 | 9.70–9.92 |
| Visual construction |  |  |  |  |  |  |  |  |  |
|  | Model 1 | 9.78 | 9.68–9.88 | 9.74 | 9.64–9.84 | 9.88 | 9.78–9.98 | 9.81 | 9.71–9.91 |
|  | Model 4 | 9.80 | 9.67–9.93 | 9.77 | 9.63–9.90 | 9.90 | 9.77–10.03 | 9.82 | 9.69–9.96 |
| Fluency |  |  |  |  |  |  |  |  |  |
|  | Model 1 | 8.65 | 8.44–8.87 | 8.76 | 8.55–8.98 | 8.99 | 8.77–9.21 | 8.68 | 8.46–8.90 |
|  | Model 4 | 8.63 | 8.35–8.91 | 8.77 | 8.48–9.05 | 8.99 | 8.70–9.27 | 8.67 | 8.37–8.96 |
|  | Model 1 | 9.49 | 9.26–9.73 | 9.43 | 9.19–9.66 | 9.66 | 9.43–9.90 | 9.34 | 9.11–9.58 |
|  | Model 4 | 9.31 | 9.01–9.61 | 9.28 | 8.97–9.59 | 9.51 | 9.20–9.82 | 9.17 | 8.86–9.49 |

CASI, Cognitive Ability Screening Instrument; VSR, area of abdominal visceral adipose tissue to area of abdominal subcutaneous tissue ratio; CI, confidence interval.

Model 1 was adjusted for age and years of education.

Model 4 was adjusted for the variables in Model 3 plus hypertension (yes or no), diabetes (yes or no), and dyslipidemia (yes or no).

No significant differences were observed among VSR quartiles.
